# Turbulence mapping: a new CMR approach for assessment of aortic stenosis

**DOI:** 10.1186/1532-429X-15-S1-P110

**Published:** 2013-01-30

**Authors:** Petter Dyverfeldt, Michael D Hope, Elaine E Tseng, David Saloner

**Affiliations:** 1 University of California San Francisco, San Francisco, CA, USA; 2 Linköping University, Linköping, Sweden

## Background

Pressure loss estimation based on the simplified Bernoulli equation frequently misclassifies the severity of aortic stenosis. Consequently, several investigators have on the basis of fluid dynamics theory derived pressure loss indices aimed at improving the clinical approach to pressure estimation [1-3]. However, CMR may offer a stronger alternative. The primary cause of pressure loss in aortic stenosis is dissipation of turbulent kinetic energy (TKE) into heat. New CMR methods permit direct estimation TKE [4]. We sought to evaluate the relationship between CMR-measured TKE and previously described pressure loss indices.

## Methods

27 patients under evaluation for aortic valve replacement were enrolled. The patient population represents a broad range of aortic stenosis and aortic dilation. Aortic valve area was 1.6 ± 1.7 cm^2^ (mean ± std dev), range: 0.4 - 6.4 cm^2^. Peak velocity was 3.7 ±1.4 m/s, range 2.0 - 7.5 m/s. Maximum aortic diameter was 4.1 ± 0.8 cm, range 3.0 - 6.1 cm.

TKE was estimated using a novel 4D Flow CMR method, as described in [4]. A measure of the total TKE (TKE_tot_) was obtained by integrating the TKE per voxel across the ascending aorta. Each subject had clinical echocardiography and computed tomography studies done close to the CMR study.

**Figure 1:**
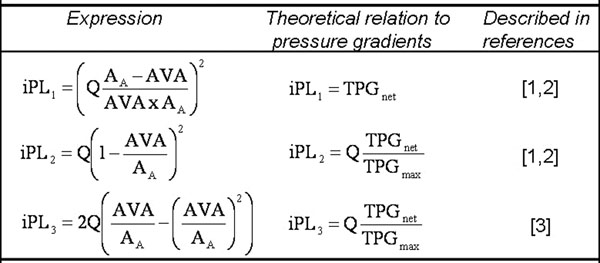
Table of pressure loss indices (iPL) used in this study, along with references from which they were derived. *TPG_max_ and TPG_net_ denote maximum and net transvalvular pressure gradient, respectively. Q = peak systolic flow rate, AVA = aortic valve area, A_A_ = aortic area at sinotubular junction. Q, AVA and A_A_ were obtained from MRI, echocardiography and computed tomography, respectively

TKE_tot_ was compared against three pressure loss indices (iPL) derived from the literature [1-3] (see table in figure 1). These indices represent different approaches to estimate pressure loss effects based on data obtainable with noninvasive imaging. The underlying theory has been compared favorably with catheter-based measurements in select settings [1-3].

## Results

**Figure 2:**
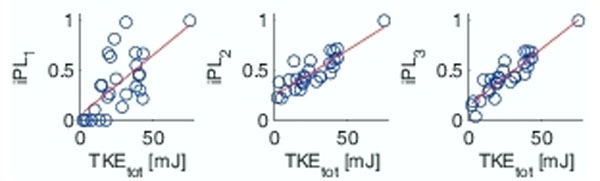
Total turbulent kinetic energy (TKE_tot_) in the ascending aorta plotted against the pressure loss indices (iPL) described in Table 1. All indices were normalized to have a maximum value of iPL = 1. TKE_tot_ was obtained by integrating the TKE per unit volume across the entire ascending aorta. Solid line: estimated regression line.

Scatter plots for TKE_tot_ vs iPL_1_, iPL_2_, and iPL_3_ are shown in Figure 2. Correlation for TKE_tot_ vs iPL_1_, TKE_tot_ vs iPL_2_, and TKE_tot_ vs iPL_3_ was 0.65, 0.87, 0.92, respectively. The slope was significantly different from zero in all cases.

## Conclusions

This study used a novel CMR flow imaging method to measure the total TKE in the ascending aorta of patients with aortic stenosis. Strong correlation was found between TKE and pressure loss indices derived from fluid dynamics theory. By directly measuring the source of irreversible pressure loss, TKE mapping allows new avenues for evaluation of aortic stenosis with CMR. Future work will include a comparison between CMR-measured TKE and catheter-based pressure measurements.

## Funding

Fulbright Commission

Swedish Heart-Lung Foundation

Swedish Brain Foundation
